# ‘Greenwashing’ tobacco products through ecological and social/equity labelling: A potential threat to tobacco control

**DOI:** 10.18332/tpc/99674

**Published:** 2018-11-16

**Authors:** Frank Houghton, Sharon Houghton, Diane O’ Doherty, Derek McInerney, Bruce Duncan

**Affiliations:** 1 Department of Applied Social Sciences, Limerick Institute of Technology, Moylish, Limerick, Ireland; 2 University of Limerick, Limerick, Ireland; 3 Hauora Tairawhiti, Gisborne, New Zealand

**Keywords:** tobacco, greenwashing, ecological labelling, environment, advertising, marketing

## Abstract

There has been significant growth in ecological/environmental labelling of products and services internationally in recent years. Such efforts have become an integral element of the marketing strategies used by many firms. Concerns have been raised, however, that for some companies, this is little more than ‘greenwashing’, i.e. a cynical attempt to boost sales without any meaningful underlying sensitivity or change, in practice. Given the extremely negative track record of the global tobacco industry *(Big Tobacco)*, it is essential that health policy makers and anti-smoking campaigners closely monitor this industry’s attempts to exploit both growing environmental concerns among consumers and gaps in legislation. Although there is relatively strong legislation in some countries, to prohibit suggestions that cigarettes may be environment friendly, a further tightening of legislation is required.

## COMMENTARY

Tobacco related deaths continue to constitute the largest cause of preventable mortality globally^1,2^. Estimates of annual global deaths from tobacco related disease have grown from six million to seven million, with projections of eight million by 2030^3^. Such estimates routinely underestimate the actual impact of tobacco, as they ignore both morbidity and the financial consequences associated with smoking, as well as the opportunity cost of tobacco production and purchase^4,5^.

However, one aspect of the global tobacco industry that is often underplayed is its adverse environmental impact^6,7^. Although health researchers routinely understand this in terms of seconhand^8^ and thirdhand smoke^9,10^, there is a wider environmental context to such discussions. Significant adverse environmental impacts of the tobacco industry involve: fertilizer and pesticide use; deforestation; water use; waste^11^; transportation and pollution; and packaging^6,12^. Particular attention from an environmental perspective has focussed on the impact of cigarette butts^13-16^. The adverse impact of the global tobacco industry has been addressed repeatedly by the World Health Organisation (WHO)^6,17^, and is specifically addressed in Article 18 of the Framework Convention on Tobacco Control (FCTC)^18^. Recent attempts to quantify the significant negative impact of the global tobacco industry include an assessment by Zafeiridou et al. on behalf of the WHO Framework Convention on Tobacco Control (FCTC)^17^. This research indicates that globally *‘the tobacco supply chain contributes almost 84 Mt CO_2_ eq emissions to climate change, 490000 tonne 1,4-DB eq to ecosystem ecotoxicity levels, over 22 billion m3 to water and 21 Mt oil eq to fossil fuel depletion annually’*^17^. Given increasing environmental controls in many Western countries, there has been a move to production in poorer, less regulated countries in recent years^17,18^.

The global tobacco industry *(Big Tobacco)* has been likened to the many-headed hydra of ancient Greek mythology^19^. According to legend, as one snakelike head was cut-off, two immediately sprouted to replace it. Given the increasing regulation of tobacco in many Western countries (excluding the USA)^20,21^, and given their history of corporate malfeasance^22-33^, it seems extremely likely that *Big Tobacco* will exploit any potential tactic to maintain or increase sales^34-36^. This Machiavellian approach to marketing exhibited by *Big Tobacco* may be significant in the context of the growth in identity/values-based purchasing and investment in recent years, which has resulted in a significant increase in value-based labelling of goods and services^37-40^. This may be seen, perhaps most publicly, in certain aspects of the global fashion industry^41-44^. Such labelling usually covers the domains of social justice, animal welfare or environmental/sustainability issues^45^. For example, social-justice/equity-based labelling would include well known labelling such as Fair Trade^46^, as well as GoodWeave^47^, Child Labor Free^48^, RugMark^49^, and Conflict Free Diamonds^50-52^. Animal welfare labelling would include labels such as Free Range^53^, The Body Shop (‘cruelty free and forever against animal testing’)^54^ and Dolphin-friendly tuna^38,39^. Examples of environmental and sustainability labels would include Organic^55^, GMO Free^56^, Sustainably Sourced^57^, and Recyclable/made-from-recycled-materials^58^.

In assessing the potential appeal and impact of such labelling, it is vitally important to note the impact of branding. *Big Tobacco* has long understood this, and hence it is perhaps no surprise that these firms, combined, spend 26 million dollars per day in marketing^59^. Research demonstrates that smokers ascribe characteristics to known brands and types of cigarettes^60-62^, even when unknowingly comparing identical products^60^.

Concerns about *Big Tobacco* starting to exploit such concerns through ‘greenwashing’ are well founded. Evidence already exists of the industry exploring Social Life Cycle Assessment^63^, and the apparent engagement of the industry with Corporate Social Responsibility (CSR) has drawn many negative comments^64-67^. Chapman has written eloquently about *Big Tobacco’s* apparent engagement with CSR using the metaphor of a wolf in sheep’s clothing^64^. It is interesting to note that Chapman is not alone in his cynical evaluation of tobacco companies use of CSR^65^, with the World Health Organization going so far as to declare that there is an inherent contradiction between tobacco companies and corporate social responsibility^67^.

The best current example of attempts to describe cigarettes as ‘natural’ and ‘organic’ is probably the case of the Santa Fe Natural Tobacco Company, which produces Natural American Spirit cigarettes. In early 2017, the company was required to remove the terms ‘additive-free’ and ‘natural’ from its marketing materials by the US Food and Drug Administration (FDA). However, the producer is still able to retain the use of the word ‘Natural’ in its brand name. In addition, the company is still able to use the term ‘organic’ in its marketing, as well as implying the ‘healthy’ nature of its product through the use of its ingredients list, viz. ‘Tobacco Ingredients: Tobacco and Water’^68^.

Current European Union legislation explicitly prevents the marketing of tobacco products as environment friendly^20^. However, the threat remains of a tobacco company developing a brand and/or a variant name that implies an enhanced environmental sensitivity. At present, even under plain packaging legislation, tobacco manufacturers are legally still entitled to display the Brand name of their product and then on the line underneath the Variant name. A tobacco manufacturer seeking to market their ‘green’ credentials could therefore create a brand called for example ‘Mother Earth’ (English) or its equivalents ‘Gaia’ (Greek), ‘Terra’ (Roman), or ‘Jord’ (Norse). To further promulgate an environment-friendly image, variant names such as ‘Green Leaf’, ‘Blossom’ or ‘Eco’ could be used.

However, even within the European Union, current tobacco-control legislation does not cover social/equity-based labelling. Thus, *Big Tobacco* could potentially include logos or launch new brands and variants of cigarettes focusing on issues such as fair wages, or possibly social investment in schools or health systems for workers, their families and communities. This could essentially be akin to current Fair Trade marketing. Even in countries that have introduced plain packaging legislation, a tobacco firm seeking to exploit this avenue of promotion could therefore potentially create a brand called something akin to ‘Workers Paradise’ with brand variations such as ‘Equity’, ‘Solidarity’ or ‘Comrade’. It should be noted that a significant increase in Variant names on cigarettes was noted in Australia after the introduction of plain packaging^69,70^. Similarly, tobacco firms in the US have been observed to introduce Variant names in an effort to exploit loopholes in the FDA ban on terms such as ‘light’ and ‘mild’. Firms are known to use colour-based terms, such as silver to indicate light-and mild-strength cigarettes^71^.

It is highly unlikely that the Brand and Variant names alone would have a significant impact on the marketing success of such initiatives. However, although strict controls on tobacco advertising and sponsorship exist in countries such as Australia, New Zealand and Member States of the European Union, control of online and social media marketing may be an Achilles heel in such regulation, particularly given the significant growth in the average amount of time people spent using such technologies^72-74^. National borders and local legislation mean little in an increasing online and virtual world.

In relation to the evolving and deceptive nature of tobacco marketing, it is important to remember the Marlboro Formula 1 (F1) logo controversy. Philip Morris began funding the Ferrari F1 team in 1968 to promote the Marlboro brand^75,76^. After many years of significant and public funding, the 2005 European Union Tobacco Advertising Directive prohibited such overt sponsorship, in line with recommendations of the Framework Convention on Tobacco Control (FCTC). However, funding by Philip Morris continued, and although the Marlboro logo ostensibly disappeared, it continued covertly with a ‘Barcode’ design that has been neatly described as a form of ‘alibi’ marketing. Although Ferrari claimed that the new design was simply part of the livery of the car, it was obvious that the location, colours and design of the logo could not fool anyone and was eventually withdrawn^75^. Tobacco industry funding of the team has continued and it could justifiably be argued that the red Ferrari F1 car and Marlboro are currently synonymous to many older smokers.

The creativity of firms to circumvent bans on advertising is also evident in the parallel sector of alcohol control. This is evident in tactics adopted by the Welsh brewery Brains, which has sponsored the Welsh rugby team since 2004. In 2005, in response to French legislation that prohibits alcohol sponsorship of televised games played on French soil, the ‘Loi Évin’, the Welsh team played in jerseys reading ‘Brawn’ rather than ‘Brains’. In 2007, the club again played in France, this time sporting jerseys reading ‘Brawn Again’^77,78^.

## CONCLUSION

Regulation of tobacco remains weak in many countries, including leading economies such as China and the USA. In such loosely regulated environments, the potential for the adoption of ecological and social/equity-based marketing is obvious. In countries with stricter legislation there is no room for complacency. The tobacco industry is well known for its ingenious marketing techniques and its hydra-like ability to respond aggressively to attempts to rein in and control its activities. It is imperative, therefore, that legislation is introduced to prevent exploitation of loopholes.
